# 3D printed fracture reduction guides planned and printed at the point of care show high accuracy – a porcine feasibility study

**DOI:** 10.1186/s40634-022-00535-2

**Published:** 2022-09-27

**Authors:** Andreas Hecker, Sophie C. Eberlein, Frank M. Klenke

**Affiliations:** Department of Orthopaedic Surgery and Traumatology, Inselspital, Bern University Hospital, University of Bern, Freiburgstrasse 4, 3010 Bern, Switzerland

**Keywords:** 3D print, 3D reconstruction, Diaphyseal fracture, Comminuted fracture, Malalignment, Malrotation

## Abstract

**Purpose:**

After surgical treatment of comminuted diaphyseal femoral and tibial fractures, relevant malalignment, especially rotational errors occur in up to 40–50%. This either results in a poor clinical outcome or requires revision surgery. This study aims to evaluate the accuracy of reduction if surgery is supported by 3D guides planned and printed at the point of care.

**Methods:**

Ten porcine legs underwent computed tomography (CT) and 3D models of femur and tibia were built. Reduction guides were virtually constructed and fitted to the proximal and distal metaphysis. The guides were 3D printed using medically approved resin. Femoral and tibial comminuted diaphyseal fractures were simulated and subsequently reduced using the 3D guides. Postoperative 3D bone models were reconstructed to compare the accuracy to the preoperative planning.

**Results:**

Femoral reduction showed a mean deviation ± SD from the plan of 1.0 mm ± 0.9 mm for length, 0.9° ± 0.7° for varus/valgus, 1.2° ± 0.9° for procurvatum/recurvatum and 2.0° ± 1.7° for rotation. Analysis of the tibial reduction revealed a mean deviation ± SD of 2.4 mm ± 1.6 mm for length, 1.0° ± 0.6° for varus/valgus, 1.3° ± 1.4° for procurvatum/recurvatum and 2.9° ± 2.2° for rotation.

**Conclusions:**

This study shows high accuracy of reduction with 3D guides planned and printed at the point of care. Applied to a clinical setting, this technique has the potential to avoid malreduction and consecutive revision surgery in comminuted diaphyseal fractures.

**Level of Evidence:**

Basic Science.

## Introduction

Due to the individual anatomy, orthopedic trauma surgery, like many other disciplines, is more and more moving towards individualized and therefore more precise medicine. This aims to improve the individual outcome for the patient.

The 3D planning of surgeries and their execution with 3D printed templates adapted to the individual patient is already an established procedure for planned surgeries such as corrective osteotomies for mal-united fractures [11, 23]. Currently, the planning and production of 3D templates is mostly performed by external companies. The duration until delivery usually exceeds 4 weeks. Therefore, it is not possible to use them in acute cases (e.g. acute fractures) that need to be treated surgically within a few days.

The most frequent postoperative deformity in long bone shaft fractures is malrotation [1]. In femoral fractures, rotational errors occur in up to 40% of cases after surgical treatment, which either results in a poor clinical outcome or requires revision surgery [6, 10]. In tibial diaphyseal fractures, up to 50% rotational errors have been reported [4, 7]. The higher the degree of comminution the higher are the chances of malrotation postoperatively. The reason is, that due to the comminuted situation, no reliable bony references exist intraoperatively and the surgeon can only estimate the correct length, rotation, and coronal/sagittal alignment based on the contralateral leg [7].

The exact definition of a malrotated femur or tibia remains controversial. Tolerances range between 10 and 30° in the femur [1, 2, 6] and 10–25° in the tibia [7, 25]. It was shown that femoral rotational malalignment compared to the contralateral side of ≥10° was likely to be symptomatic and that hip, knee, and patellofemoral joints were affected [15]. Large degrees of lower extremity malrotation lead to a higher incidence of hip, knee and ankle osteoarthritis [9]. The other dimensions of malalignment (length and sagittal/coronal axis) have also been shown to frequently cause clinical symptoms [28] and premature degeneration [12, 13].

This project’s aim was to assess the accuracy of 3D planned surgery with surgical guides planned and printed at the point of care in a porcine cadaver fracture setting. The long-term goal is to use this technique in the acute treatment of complex fractures and thus reduce the above-mentioned complications.

We hypothesized that length, axis and rotation of the porcine femur and tibia have a mean deviation from the plan of less than 3 mm (length), 3° (coronal and sagittal axis) and 5° (rotation) after fixation of a simulated comminuted shaft fracture using 3D printed guides.

## Methods

Ten mature (age > 2 years) porcine leg bones were used for this study. The cadaveric bones underwent computed tomography (CT) (SOMATOM X.cite, Siemens Healthcare GmbH, Eschborn, Germany) in a high quality of 0.5 mm slices.

### Planning

**Fig. 1:**
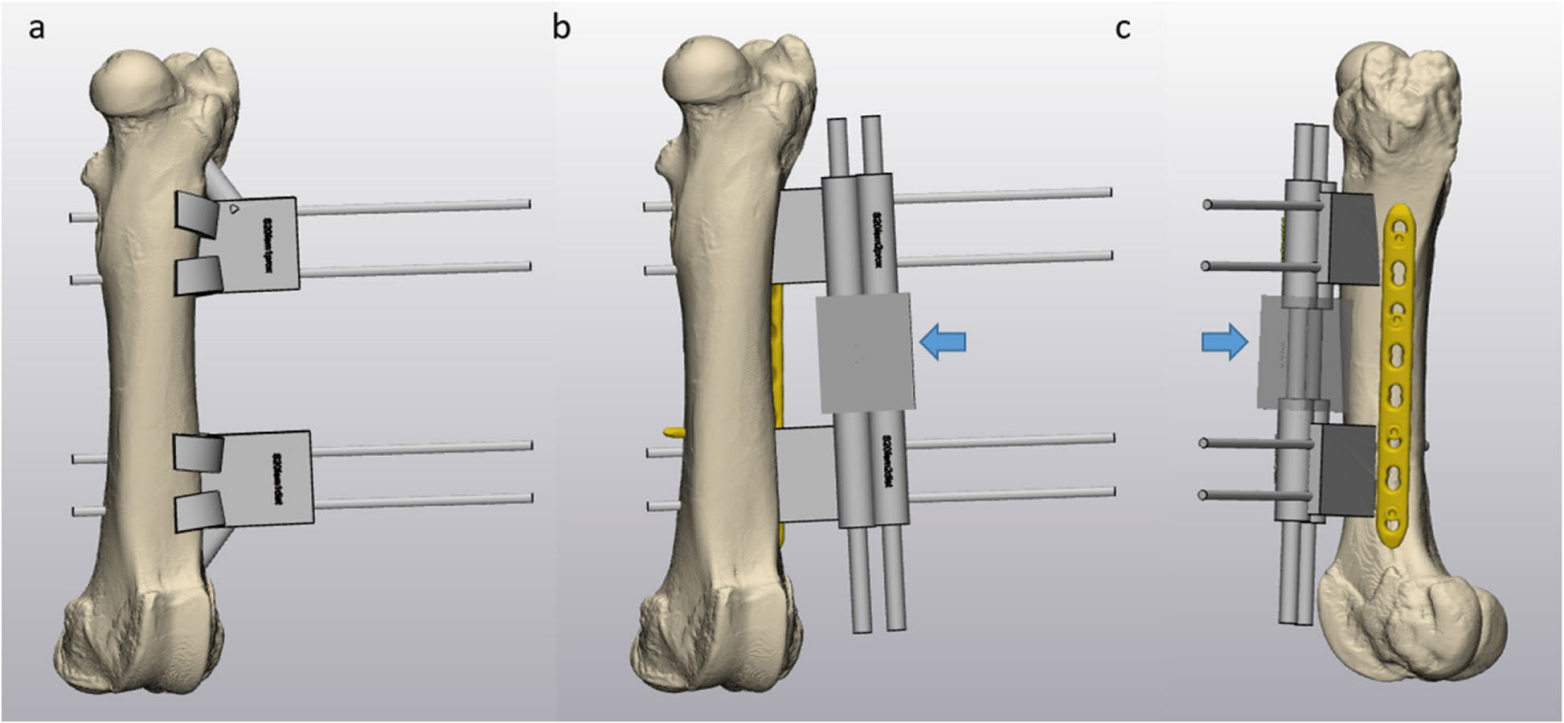
Femoral surgical guide planning with Materialise 3-matic software. **a** shows the first step guides with additional “feet” to allow proper bone contact and high accuracy in anteromedial view. Drill pins are inserted through these guides and define the fragment in space. Second step connection guides were slid over these drill pins consecutively. They were slimmer and allowed plate placement, as displayed anteromedial view (**b**) and lateral view (**c**). To connect the proximal and distal second step guide, a reduction/connection guide was used in-between (blue arrow)

**Fig. 2:**
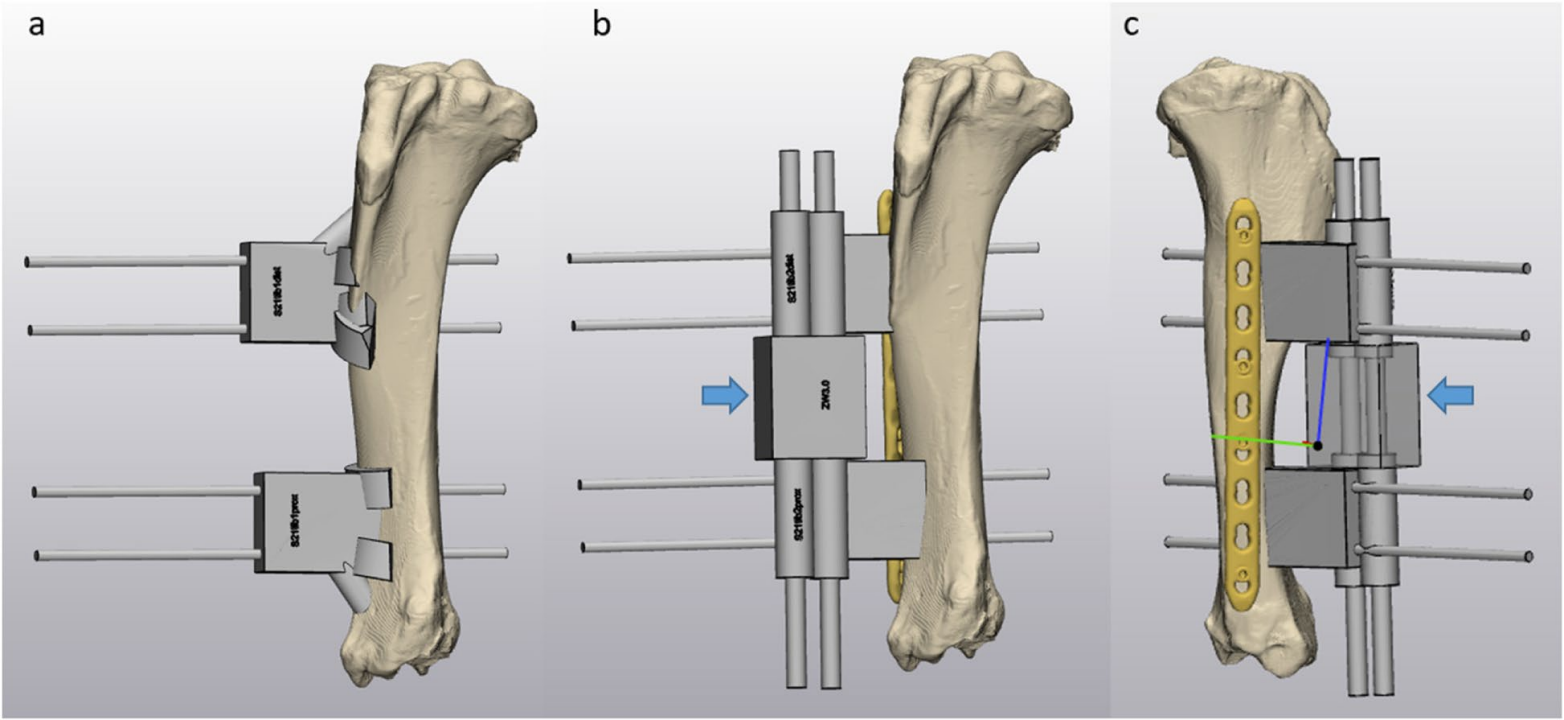
Tibial surgical guide planning with Materialise 3-matic software. **a** shows the first step guides with additional “feet” to allow proper bone contact and high accuracy in anterolateral view. Drill pins are inserted through these guides and define the fragment in space. Second step connection guides were slimmer and allowed plate placement, as displayed anterolateral view (**b**) and medial view (**c**). To connect the proximal and distal second step guide, a reduction/connection guide was used in-between (blue arrow)

These 2D sectional images were segmented and individual 3D models were reconstructed using the medically certified software “Mimics 23.0” (Materialise GmbH, Munich, Germany). Based on these 3D models, virtual surgical guides were designed and fitted to one exact position on the bone by Boolean operations using “3-matic 14.0” (Materialise GmbH, Munich, Germany). A key factor for exact positioning of the guide is a three-point support. A metaphyseal support structure (Figs. 1a and 2a) guarantees length control. Anterior and posterior supports covering at least one prominent edge or bone prominence are crucial for rotational fit. First, one guide was planned proximal and distal to a simulated shaft fracture. Subsequently, to connect the proximal and distal femur in the desired anatomic position, a connecting reduction guide was created, that included a cylindrical recess for carbon fiber rods (diameter 8 mm, Stryker, Kalamazoo, Michigan, USA). The latter were meant to strengthen the construct. Guide planning is displayed in Figs. 1 and 2.

### 3D printing and processing

The guides were printed with a professional 3D printer certified for medical applications (Form 3B, Formlabs, Berlin, Germany). Filling material was BioMed Amber Resin (Formlabs, Berlin, Germany), also approved for medical applications and for autoclave sterilization. After printing, the parts underwent isopropanol washing (Form Wash, Formlabs, Berlin, Germany) for 20 minutes, drying at 50 °C for 30 minutes (dehydrator, kitchtrixx GmbH, Berlin, Germany) and ultra-violet (UV) light curing (Form Cure, Formlabs, Berlin, Germany) for 60 minutes. As there are supporting structures necessary for printing, they were removed with side cutters after this processing step. Object orientation during printing was chosen in a way, that no support structures touched the area with planned bone contact. Sterilization was performed following our in-house autoclave protocol for 18 minutes at 134 °C (Euro Selectomat, MMMGroups GmbH, Munich, Germany). Time from CT scan of the bone to the 3D printed guides being ready for usage did not exceed 24 hours for one bone.

### Surgical technique

**Fig. 3:**
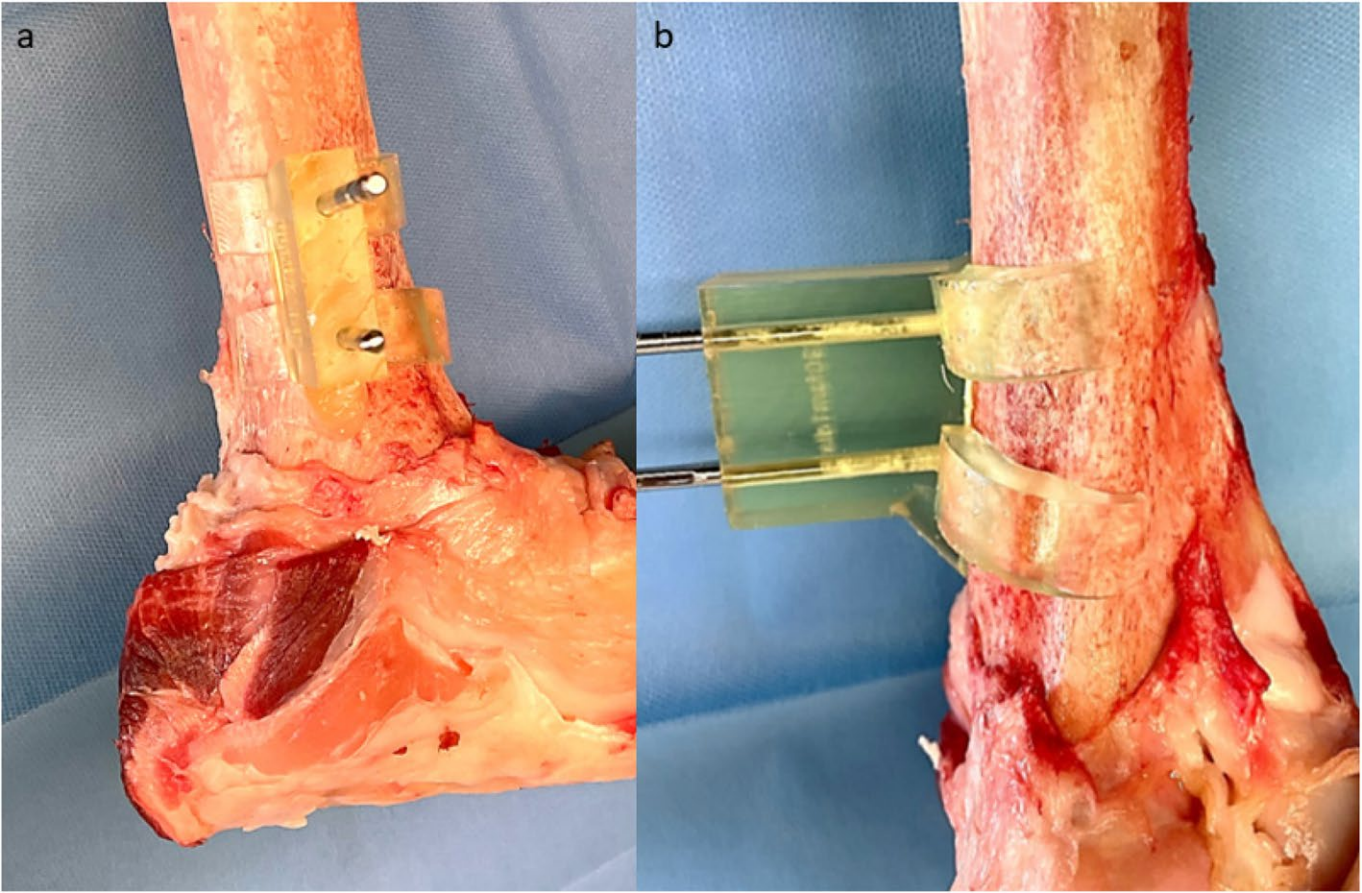
Femoral surgical site with fitted guide. **a** shows the first guide fitted to the lateral aspect of the distal femur, fixed with Schanz pins in lateral view. **b** demonstrates a closer anteromedial view of the anatomical shaped guide

**Fig. 4:**
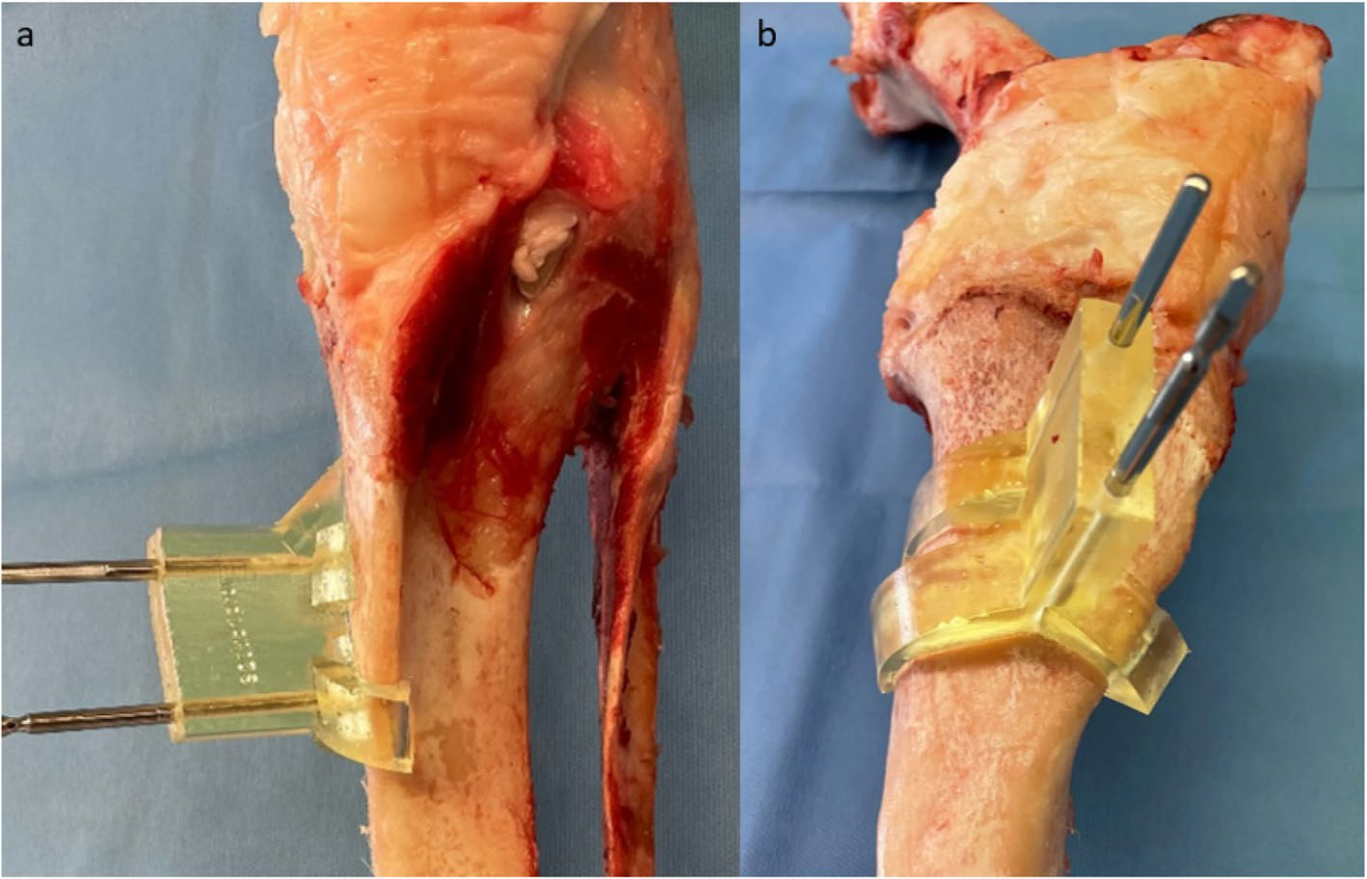
Tibial surgical site with fitted guide. **a** shows the first guide fitted to the medial proximal tibia, fixed with Schanz pins in frontal view. **b** demonstrates a closer anteromedial view of the anatomical shaped guide

Subsequently, surgical preparations were performed. To simulate a diaphyseal comminuted fracture zone without bony references and no contact of the proximal and distal main fragment, 2 cm bone was resected within the diaphysis of tibia and femur. To apply the first step surgical guides directly to the bone, very accurate dissection was needed. The periosteum was elevated off the contact areas of the guides. With respect to their corresponding surface, the guides were applied to the proximal and distal femur and tibia and drill pins (Schanz pin, diameter 4 mm, Stryker, Kalamazoo, Michigan, USA) were inserted for fixation, as shown in Figs. 3 and 4.

As there was hardly soft tissue left on the bones, there was no need for any surgical approach. However, placement of guides and plate fixation was chosen to fit for a lateral subvastus approach to the femur and an anteromedial approach to the tibia.

**Fig. 5:**
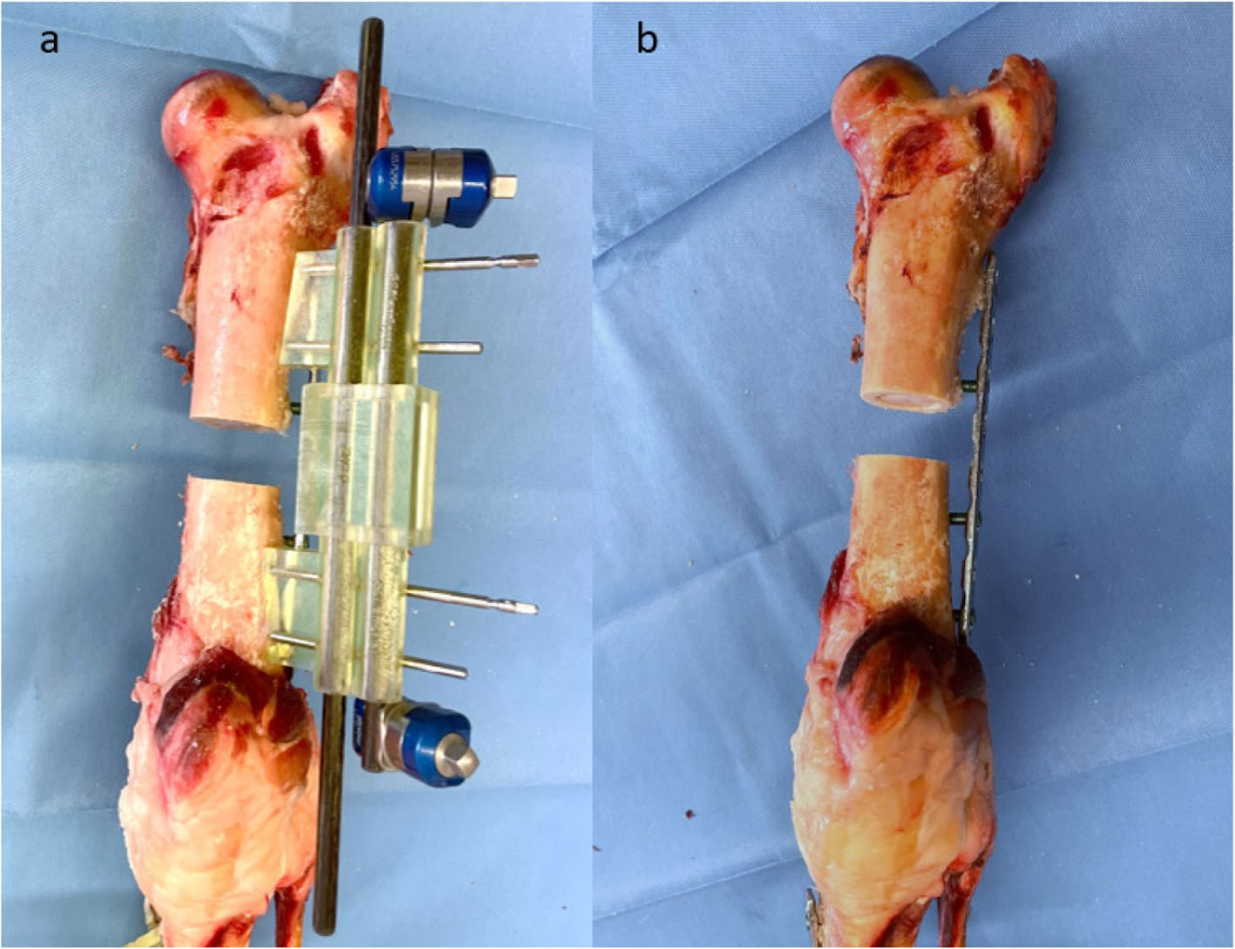
Femoral surgical site after reduction and plate fixation. **a** displays a frontal view of the femoral surgical site after defect creation, reduction and plate fixation with the reduction guides and bridging carbon fiber rods still in place. **b** shows the final result with the reduction guides and pins removed

**Fig. 6:**
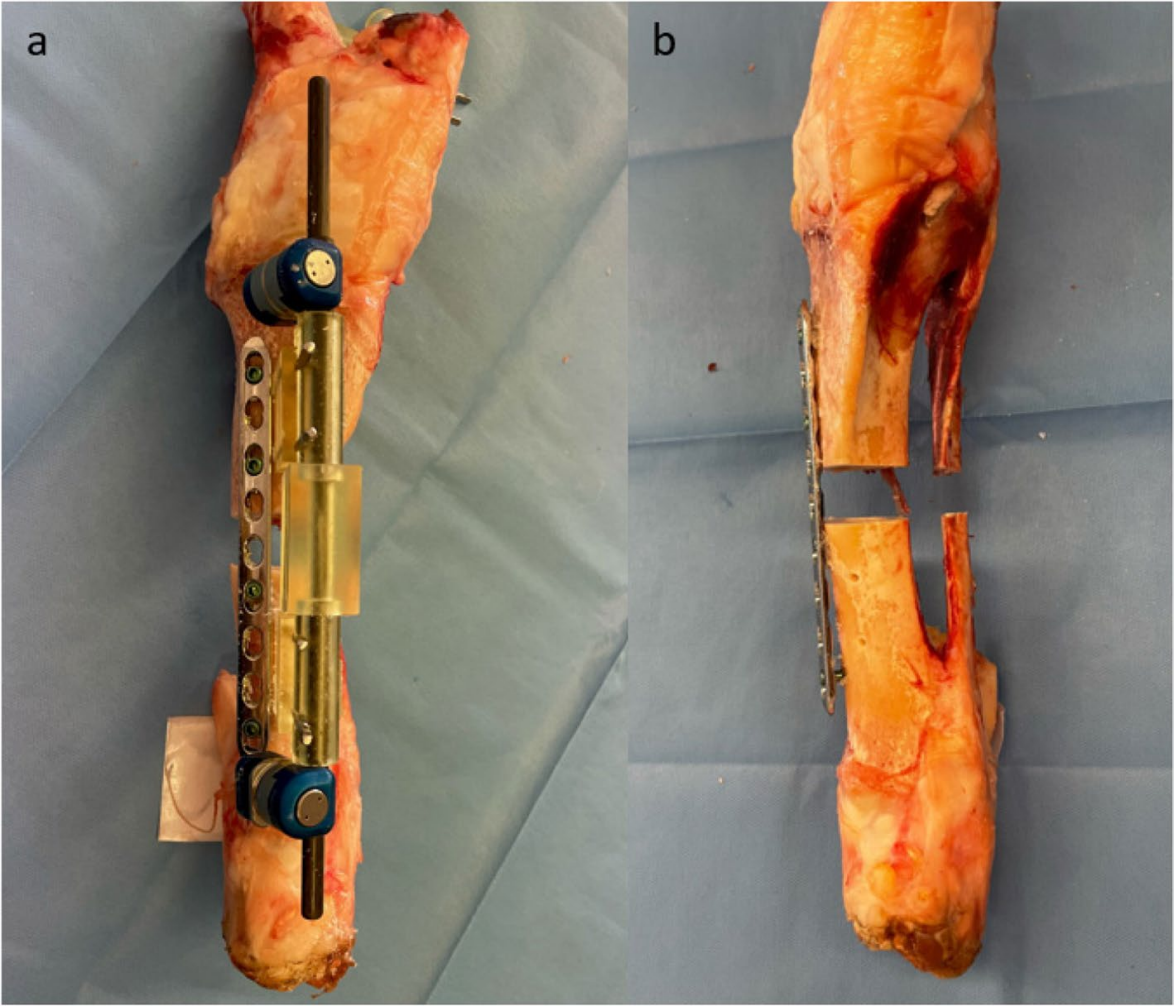
Tibial surgical site after reduction and plate fixation. **a** shows the anteromedial view after plate fixation with the reduction guides and bridging carbon fiber rods still in place. **b** shows final result with the reduction guides and pins removed in the frontal view

With the help of the second step reduction guides slid over the distal and proximal drill pins, and the bridging guide in-between, the construct was connected. These guides could only be fitted together in the planned position, which automatically led to reduction of the proximal and distal fragments. Stability was improved by addition of two carbon fiber rods bridging the construct. Subsequently, the achieved position was held by attachment of an angle stable surgical plate and screws (4.5/5.0 mm stainless steel LCP, Depuy Synthes, Solothurn, Switzerland) and the reduction guides were removed. Reduction and fixation are displayed in Figs. 5 and 6.

### Alignment analysis

**Fig. 7:**
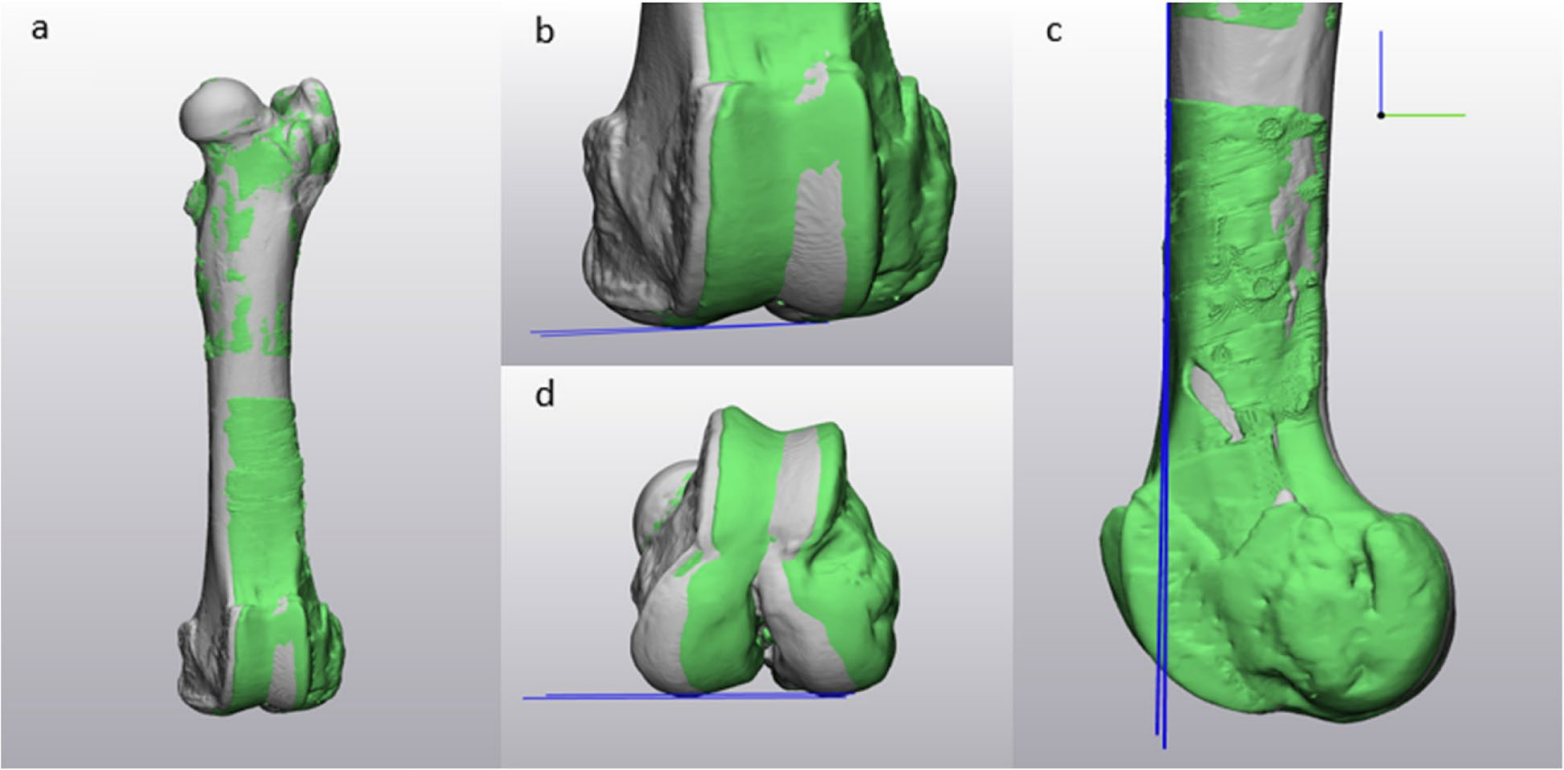
Comparison of postoperative result to the preoperative plan of the femur with Materialise 3-matic software. Each picture shows two porcine femoral bone models. The preoperative model is grey while the achieved result is green. Both models were fitted proximally (automatic registration, **a**). Subsequently, the measurements of varus/valgus (**b**), procurvatum/recurvatum (**c**) as well as rotation (**d**) were performed distally. The blue lines indicate a tangent to each of the bone at defined points. For varus/valgus and rotation a tangent to the most distal point of the medial and lateral distal femoral condyle was drawn. For procurvatum/recurvatum, the tangent was fitted to the anterior femoral cortex on a lateral view. The angle between these lines represents the respective deviation from the plan

**Fig. 8:**
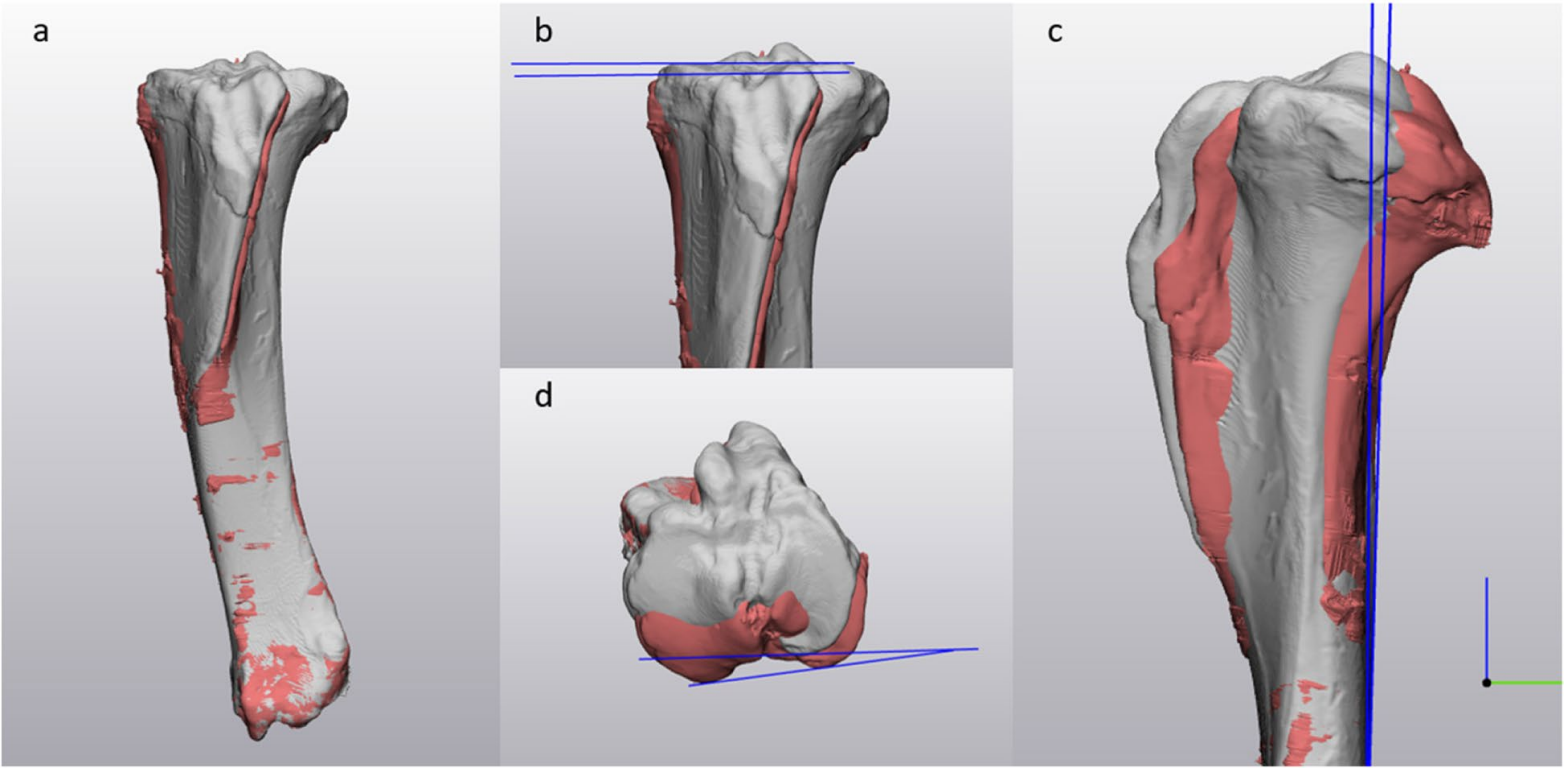
Comparison of postoperative result to the preoperative plan of the tibia with Materialise 3-matic software. Each picture shows two porcine tibial bone models. The preoperative model is grey while the achieved result is green. Both models were fitted distally (automatic registration, **a**). Subsequently, the measurements of varus/valgus (**b**), procurvatum/recurvatum (**c**) as well as rotation (**d**) were performed proximally. The blue lines indicate a tangent to each of the bone at defined points. For varus/valgus and rotation a tangent to the most proximal point of the medial and lateral tibial plateau was drawn. For procurvatum/recurvatum, the tangent was fitted to the posterior tibial cortex on a lateral view. The angle between these lines represents the respective deviation from the plan

Postoperatively, the specimens underwent repeated CT imaging. Again, 3D models were created of this postinterventional situation. To accurately assess deviations, the Materialise 3-matic software allows registration of two similar 3D models to each other (global registration, min. 200 iterations). Thus, the postoperative 3D model was superimposed virtually over the preoperative model. Analyzing the femoral bone, only the proximal part of the model was registered and alignment changes measured distally. Analyzing the tibial bone, models were exactly superimposed distally and measurement took place proximally. Determined reduction parameters were rotation, length and axis (varus-valgus, procurvatum/recurvatum). “Datum planes” perpendicular to coronal, sagittal and axial views were created at defined bony landmarks. For femoral measurements, a tangential plane to the distal (varus/valgus) and posterior (rotation) condyles and anterior femoral cortex (procurvatum/recurvatum) were created. For tibial measurements the planes were created tangential to the tibial plateau (varus/valgus), the posterior tibial condyles (rotation) and the posterior tibial cortex (procurvatum/recurvatum). Deviating angles were assessed by plane-to-plane measurement, as displayed in Figs. 7 and 8.

### Statistical analysis

SPSS statistics was used for data analysis (IBM SPSS Statistics, Version 25 for Windows). According to Kolmogorov-Smirnov-Test data was normally distributed and is given as mean and standard deviation (SD). Deviations from the plan are given in millimeters (mm) for length and degrees (°) for coronal/sagittal axis and rotation. The study questions were answered by using simple descriptive statistics.

## Results

Virtually superimposed pre- and postoperative 3D bone models were analysed. Based on absolute values (regardless of the direction of the deviation from the plan), analysis revealed the following: Femoral reduction showed a mean deviation ± SD from the plan of 1.0 mm ± 0.9 mm for length, 0.9° ± 0.7° for varus/valgus, 1.2° ± 0.9° for procurvatum/recurvatum and 2.0° ± 1.7° for rotation.

Analysis of the tibial reduction revealed a mean deviation from the plan of 2.4 mm ± 1.6 mm for length, 1.0° ± 0.6° for varus/valgus, 1.3° ± 1.4° for procurvatum/recurvatum and 2.9° ± 2.2° for rotation.

Tables 1 and 2 show the mean residual errors considering the different directions (shortening/lengthening, varus/valgus, procurvatum/recurvatum, and internal/external rotation) of deviation.

**Table 1:** Results of the femoral 3D alignment analysis

	Direction of deviation	n	Deviation in millimeters/degree
Length	all	10	1.0 mm ± 0.9 mm
	shortening	2	1.1 mm
	lengthening	8	1.0 mm
a.p. axis	all	10	0.9° ± 0.7°
	varus	4	0.8°
	valgus	6	0.9°
Lateral axis	all	10	1.2° ± 0.9°
	procurvatum	4	1.3°
	recurvatum	6	1.1°
Rotation	all	10	2.0° ± 1.7°
	internal rotation	5	1.6°
	external rotation	5	2.4°

Different overall deviations are given as well as subgroups indicating the direction of malalignment. Column *n* demonstrates the number of cases. Overall values are given as mean ± standard deviationin millimeters and degrees

**Table 2:** Results of the tibial 3D alignment analysis

	Direction of deviation	n	Deviation in millimeters/degree
Length	all	10	2.4 mm ± 1.6 mm
	shortening	8	2.7 mm
	lengthening	2	1.2 mm
a.p. axis	all	10	1.0° ± 0.6°
	varus	3	1.2°
	valgus	7	1.0°
Lateral axis	all	10	1.3° ± 1.4°
	procurvatum	4	1.7°
	recurvatum	6	1.0°
Rotation	all	10	2.9° ± 2.2°
	internal rotation	7	2.6°
	external rotation	3	3.5°

Different overall deviations are given as well as subgroups indicating the direction of malalignment. Column *n* demonstrates the number of cases. Overall values are given as mean in millimeters and degrees

## Discussion

This study shows that 3D planning and printing of individual reduction guides at the point of care is feasible and leads to high accuracy in simulated fracture reduction and fixation in a porcine model.

We described a technique to reduce comminuted diaphyseal fractures of long bones of the lower extremity and subsequently tested its accuracy. The obtained results fulfilled our expectations. The set hypothesis could be confirmed.

In the current literature, there exist several studies investigating the accuracy of patient-specific instruments. Regarding rotational alignment in 3D guided forearm osteotomies, a mean residual rotational error compared to the plan of 8.3° (opening wedge osteotomies) and 3.5° (closing wedge osteotomies) was observed [27]. These values clearly exceed the 2.0° and 2.2° observed in our study, although a direct comparison remains challenging due to the missing soft tissue in our setting.

Several studies reported high accuracy after 3D guided high tibial osteotomies (HTO) regarding axis alignment. Chaouche et al. presented excellent results of 100 patients with an executional accuracy of 1° ± 0.95° hip-knee-ankle (HKA), 0.54° ± 0.63 medial proximal tibia angle (MPTA), and 0.43° ± 0.8 posterior proximal tibia angle (PPTA) [5]. In comparison to our study the postinterventional analysis was performed on conventional radiographs, which might be less accurate. Other authors used 3D measurement tools based on CT images. Munier et al. for example found errors of less than 2° in the coronal and sagittal planes in 9/10 patients [19]. Fucentese et al. reported 0.1° ± 2.3° in the coronal plane, 0.2° ± 2.3° in the transversal plane, and 1.3° ± 2.1° in the sagittal plane [11], which matches our accuracy findings and may be related to the similar measurement technique. None of these studies reported postoperative length deviations. The length discrepancy of 1 mm and 2.4 mm respectively, as observed in this study, is not likely to be relevant in a clinical scenario, as this lies in the range of intra-individual variance [24, 25]. Another study group used 3D technology and customized 3D reduction guides based on an ipsilateral model in acute fracture care. They reported favorable radiological results in one case with an external reduction aid based on virtual 3D reduction of every fragment after femoral and tibial external fixator placement. A persistent femoral varus deviation of 4.7° and tibial valgus deviation of 4° was reported. Nevertheless, virtual 3D fragment reduction reaches its limits in C-type fractures with severe comminution and/or bone loss, as simulated by our model [18].

Applied to a veterinary fracture setting, Johnson et al. compared two techniques very similar to ours in canine tibiae. Both of their techniques showed minor alignment discrepancies compared to the intact tibia within clinically acceptable standards (0.8/2.3 mm for length, 1.7/2.9° for coronal plane alignment, 5.0/5.4° for sagittal plane alignment, 3.6/3.5° for torsional alignment) [14]. Despite a more accurate length restoration, the observed changes after reduction are slightly higher than the ones we reported for porcine tibiae. One cause might be the different anatomy of canine compared to porcine bones. Another cause might be the soft tissue tension, as they performed the surgeries on complete cadaveric specimens. Nonetheless, the results are comparable and support the accuracy of 3D printed fracture reduction guides. Another author published two case reports, using almost the same technique. Osteosynthesis of a humeral fracture in a cat showed restoration of near-anatomic humeral conformation and 3D-guided atlanto-axial fusion was also successfully performed in a dog [21, 22].

Considering the frequent malalignment in complex fractures, the aim of patient management should be to ‘Get It Right First Time’ [7]. 3D planning based on sectional images and customized templates proved to lead to more precise results than today’s free-hand gold standard [8, 17, 26, 29]. Overall, it might reduce malunion and/or revision surgery in comminuted diaphyseal lower extremity fractures by 30–50% [4, 6, 7, 10].

This study has some limitations. We used the ipsilateral uninjured bone as a template, which is not possible in a realistic posttraumatic setting. Many authors advocate the use of the contralateral bone as a reference for alignment [16, 24, 25]. However, the principle of intra-individual bony symmetry does not rule out individual variances [1]. Differences to the contralateral bone of 1.2 cm for femoral length and 1.0 cm to 2.1 cm for tibial length have been described [24, 25]. Torsional differences range from 10° (femoral mean difference) to 13° (femoral 99th percentile) and 5° (tibial mean difference) to 14° (tibial 99th percentile) respectively [3, 24, 25]. Therefore, using the contralateral side as template might also lead to malreduction. Nevertheless, the uninjured side is the best available reference in case of severe comminution.

In the development of this technique, we realized that guide placement was more difficult at the proximal femur and distal tibia due to the more cylindrical shape of the bone. This has been discussed for the canine tibia by Johnson et al. [14]. We mainly solved the problem with a three-point support as outlined in the methods section (Figs. 1a and 2a). In the authors’ opinion, this is a crucial design element to ensure good accuracy.

Another limitation is that soft tissue conditions were not considered. This theoretically could alter the results in a clinical setting. Despite this possibility clinical studies using bone referenced 3D guides could show a high accuracy of this technique [11]. A subsequent human cadaveric study, including established surgical approaches, will answer further questions and support optimization of the technique. In a clinical setting, the amount of elevated periosteum off the bone has to be as minimal as possible to avoid devascularisation and preserve the biology of the bone. Established surgical techniques proved that bone vascularization is still sufficient if excessive damage to the periosteum is avoided [11]. Moreover, in the presented technique, the contact surfaces of the guides do not lie in the fracture zone, where blood supply is even more important [20].

Overall, surgery with patient-specific 3D printed guides yields an auspicious option for fracture treatment. Our findings are in line with the limited available literature that reports high accuracy of these 3D applications.

## Conclusion

This study supports the feasibility and accuracy of 3D planning and manufacturing of 3D printed reduction guides at the point of care. The main advantage of this setting is the fast availability and the possibility to use 3D technology in acute fracture treatment. In addition, the surgeon is directly involved in the planning process, which can improve consideration of anatomical conditions and surgical approaches.

## Data Availability

All data analysed during this study are included in this article. All authors approved the version to be published.

## Abbreviations

2D: Two-dimensional
3D: Three-dimensional
CT: Computed tomography
SD: Standard deviation
UV: Ultra-violet
min.: Minimal
HTO: High tibial osteotomies
HKA: Hip-knee-ankle
MPTA: Medial proximal tibia angle
PPTA: Posterior proximal tibia angle
